# In this month’s Bulletin

**DOI:** 10.2471/BLT.17.000217

**Published:** 2017-02-01

In the editorial section, Piyasakol Sakolsatayadorn and Margaret Chan (86) introduce this special issue on the health of vulnerable populations. Abdul Ghaffar et al. (87) explain why health systems research needs to be done by the people who will use the results.

Shiva Raj Mishra (90–91) reports on sexual and reproductive health services and education programmes for adolescents in Nepal.

Fiona Fleck interviews Mani Jegathesan (92–93) about his experience as an Olympic athlete and his subsequent career as an advocate for public health.

## Ethiopia

### Hard times

Tefera Darge Delbiso et al. (94–102) study the associations between drought and conflict and childhood wasting and stunting.

## Brazil

### Doctors in rural areas

Leonor Maria Pacheco Santos et al. (103–112) observe the effects of policies to deploy more doctors.

## Liberia

### Community health workers

Peter W Luckow et al. (113–120) examine ways of delivering maternal and child health services

## Nepal

### Recognizing tuberculosis

Dipu Joshi et al. (135–139) trial case-finding by peers of people living with HIV.

## Thailand

### Universal health coverage – beyond citizens

Viroj Tangcharoensathien et al. (146–151) assess the introduction of health insurance for migrant workers.

### Maintaining autonomy

Sirinart Tongsiri et al. (140–145) detail efforts to adapt home environments for people with disabilities.

## Global

### Where are immunization programmes inequitable?

Catherine Arsenault et al. (128–134) create a dashboard to monitor vaccination coverage.

### Ensuring support for vulnerable populations

Daniela C Rodríguez et al. (121–127) argue for ongoing political commitment.

Rebekah Thomas et al. (154–156) list steps to ensuring the inclusion of transgender people.

### Caring for survivors

Emmanuel d'Harcourt et al. (157–158) consider how to improve the lives of people affected by conflict.

Jody-Anne Mills et al. (162–164) explain why rehabilitation services should be part of disaster response.

### Illustrations of inverse care

Devaki Nambiar & Harsh Mander (152–153) enumerate health risks faced by poor people in urban settings.

Matthew Grenall et al. (159–161) follow an evolving focus on vulnerable populations by the Global Fund to Fight AIDS, Tuberculosis and Malaria.

